# Analytical Measurements to Elucidate Structural Behavior of 2,5‐Dimethoxy‐1,4‐benzoquinone During Charge and Discharge

**DOI:** 10.1002/cssc.201903575

**Published:** 2020-04-28

**Authors:** Hikaru Sano, Nobuhiko Takeichi, Minami Kato, Masahiro Shikano, Tetsu Kiyobayashi, Hajime Matsumoto, Susumu Kuwabata, Masaru Yao

**Affiliations:** ^1^ Research Institute of Electrochemical Energy Department of Energy and Environment National Institute of Advanced Industrial Science and Technology (AIST) 1-8-31 Midorigaoka Ikeda Osaka 563-8577 Japan; ^2^ Department of Applied Chemistry Graduate School of Engineering Osaka University 2-1 Yamada-oka Suita Osaka 565-0871 Japan

**Keywords:** batteries, NMR spectroscopy, Raman spectroscopy, SQUID, X-ray diffraction

## Abstract

Organic compounds as electrode materials can contribute to sustainability because they are nontoxic and environmentally abundant. The working mechanism during charge–discharge for reported organic compounds as electrode materials is yet to be completely understood. In this study, the structural behavior of 2,5‐dimethoxy‐1,4‐benzoquinone (DMBQ) during charge—discharge is investigated by using NMR spectroscopy, energy‐dispersive X‐ray spectroscopy, magnetic measurements, operando Raman spectroscopy, and operando X‐ray diffraction. For both lithium and sodium systems, DMBQ works as a cathode accompanied with the insertion and deinsertion of Li and Na ions during charge—discharge processes. The DMBQ sample is found to be in two‐phase coexistence state at the higher voltage plateau, and the radical monoanion and dianion phases have no long‐distance ordering. These structures reversibly change into the original neutral phase with long‐distance ordering. These techniques can show the charge–discharge mechanism and the factors that determine the deterioration of organic batteries, thus guiding the design of future high‐performance organic batteries.

## Graphical abstract

**Made to measure**: The crystallographic structure change of 2,5‐dimethoxy‐1,4‐benzoquinone (DMBQ) during charge–discharge is investigated by using operando X‐ray diffraction, which reveals that the DMBQ sample is found to be in two‐phase coexistence state at the higher voltage plateau. Radical monoanion and dianion phases have no long‐distance ordering for either Li or Na systems.

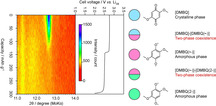

## Introduction

A recent requirement for lithium‐ion batteries is to produce a system that is free of scarce metal elements with improved battery performance. Rather than using scarce metal elements, the use of environmentally abundant metal elements, such as iron,^1^, ^2^ is a promising option. Moreover, the use of organic compounds as electrode materials is also promising.^3^, ^4^, ^5^, ^6^, ^7^, ^8^, ^9^, ^10^, ^11^, ^12^, ^13^, ^14^, ^15^, ^16^, ^17^, ^18^, ^19^, ^20^ Using charge carriers other than Li ions, such as sodium,^11^, ^21^, ^22^, ^23^, ^24^, ^25^, ^26^, ^27^, ^28^, ^29^, ^30^, ^31^, ^32^, ^33^, ^34^, ^35^ potassium,^26^ magnesium,^6^, ^36^, ^37^, ^38^, ^39^, ^40^, ^41^, ^42^, ^43^, ^44^, ^45^, ^46^, ^47^, ^48^, ^49^ calcium,^50^, ^51^ aluminum,^52^, ^53^, ^54^, ^55^, ^56^, ^57^, ^58^ or molecular ions,^59^, ^60^ is another method to develop sustainability in society. The development of systems that are completely free of scarce metal elements, including a scarce metal‐free charge carrier, will have a significant impact in a society that is heavily dependent on electricity and its consumption.

**Scheme 1:**
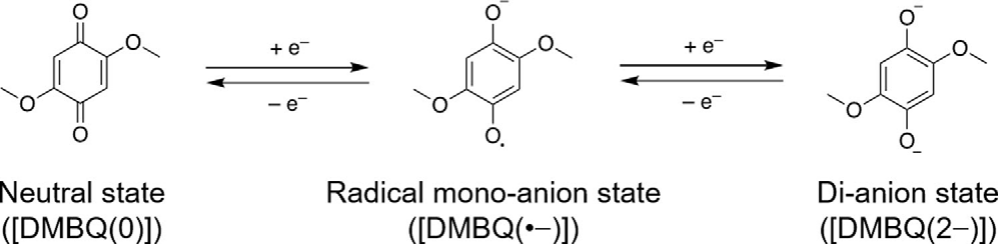
Proposed redox reaction of DMBQ during charge–discharge. DMBQ undergoes a two‐stage redox reaction.

**Figure 1:**
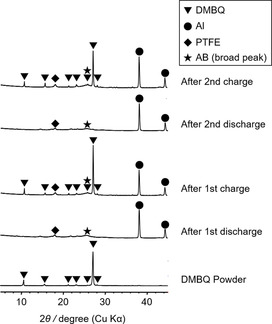
Changes in the XRD patterns of DMBQ powder and the electrode after the first and second discharge (lithiation) and charge (delithiation). This sample underwent discharge first because it was initially in an oxidized state. The charged patterns were adapted from our previous report^62^ with permission from Elsevier. The discharged ones were newly added.

Many organic compounds have been reported to fulfill the requirements of such a system. However, the reported data are primarily related to the electrochemical behavior of these compounds. In many cases, other physicochemical measurements were not fully undertaken. Previously, Yao and co‐workers tested many types of low‐molecular‐weight benzoquinone analogs as active materials and reported that 2,5‐dimethoxy‐1,4‐benzoquinone (DMBQ) was suitable as a high‐capacity positive electrode material.^43^, ^45^, ^61^, ^62^, ^63^, ^64^ They explained that DMBQ exhibits a high capacity of around 300 mAh g^−1^ because of the two‐electron transfer‐type redox reaction (Scheme 1). They reported that X‐ray diffraction (XRD) measurements of the DMBQ electrode gave certain peaks derived from crystalline DMBQ in a fully charged (delithiated) state.^61^, ^62^ As shown in Figure 1, the electrode at the fully discharged (lithiated) state did not show any peaks (the discharged data in Figure 1 were not published in any journal and are only mentioned in the Ref. 61 and 62).^61^, ^62^ However, DMBQ's crystallographic information during the transition state, particularly at the intermediate radical monoanion state [DMBQ(⋅−)], was unclear. Furthermore, as a physicochemical measurement for the DMBQ−Li system, other techniques compared to the preliminary XRD were not sufficiently investigated.

Furthermore, although it would have been of considerable interest to researchers, whether the low‐molecular‐weight molecules coexisted in two‐phases during reaction or rather in a homogeneous phase was not evident. Based on the assumed reaction formula shown in Scheme 1 and the plateau shown in the charge–discharge curve of the DMBQ electrode,^62^ it would have been reasonable to conclude that a two‐phase coexistence reaction had occurred. However, physicochemical evidence was still required to elucidate this reaction mechanism.

In this study, the detailed crystallographic structure change of DMBQ during charge–discharge in a Li system was investigated by using an operando XRD technique. This technique and the crystallographic change in an Na electrolyte system were recently developed by one of our co‐authors, Takeichi.^65^ In addition to XRD measurements, to determine the charge carrier for the DMBQ cell, ^7^Li NMR spectroscopy was carried out. Furthermore, to detect the intermediate radical compound, magnetic measurements were conducted by using a superconducting quantum interference device (SQUID). Raman spectroscopy, another common tool to examine the mechanism during charge–discharge,^66^, ^67^, ^68^, ^69^, ^70^, ^71^ was used, along with the theoretical calculations result based on density functional theory (DFT), to investigate the DMBQ electrode change behavior. In this study, the techniques examined are extremely helpful for revealing the charge–discharge mechanism and for determining the factors affecting the deterioration of organic batteries, which could form the basis on which high‐performance organic batteries could be designed.

## Results and Discussion

### Li system

#### Elucidating the carrier ion

Multiple types of organic active materials have been reported to be suitable for the Li system. Their reaction mechanisms are classified into two categories: the first is in which active materials accept and release Li ions,^72^ and the second uses an anion during the charge–discharge process rather than the Li ion. The difference in mechanism can have a considerable effect on cell design when fabricating a large‐capacity cell. Therefore, analyzing and proving the mechanism becomes very important. To identify the charge carrier in the current DMBQ battery, the Li content concentration change in the electrode was examined ex situ by using ^7^Li NMR spectroscopy.

**Figure 2:**
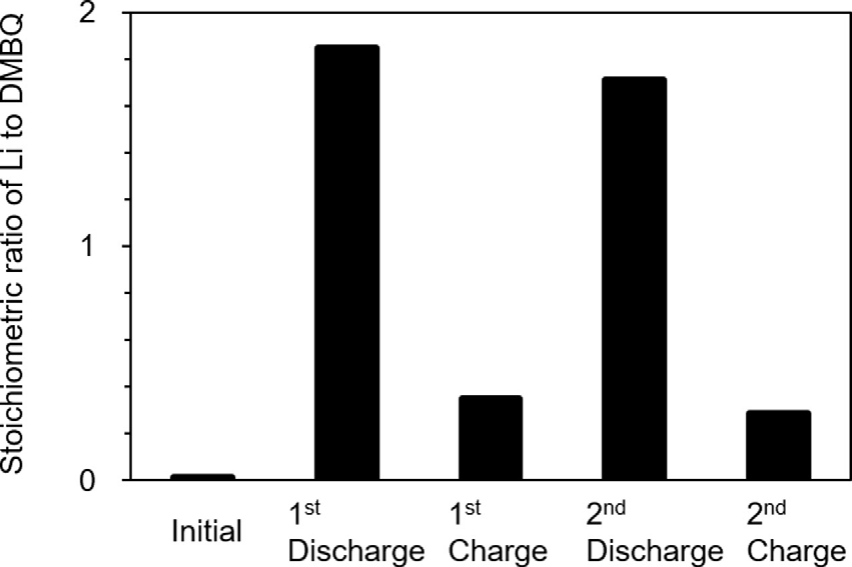
The change in the stoichiometric ratio of Li to DMBQ (*R*_Li/DMBQ_) in the extracted solutions from the electrodes in the first two cycles.

Figure 2 shows the change in the stoichiometric ratio of Li to DMBQ (*R*_Li/DMBQ_) in the extracted solutions from the electrodes in the first two cycles. While the initial DMBQ molecule did not contain any Li ions (*R*_Li/DMBQ_=0), the ratio increased to 1.8 (ca. 2) after the first discharge. Subsequently, the ratio can be reduced and become as small as 0.3 when the discharged electrode was recharged. A similar tendency in the ratio change was observed in the next cycle (second discharge: *R*_Li/DMBQ_=1.7; second charge: *R*_Li/DMBQ_=0.3). The observed stoichiometric ratio change indicated that the DMBQ molecule received and released two Li ions during the cathodic and anodic reactions and that the charge carrier in this system was considered to be a Li ion.

#### Magnetic measurement

During the charge–discharge process, the DMBQ electrode demonstrated two‐plateau potential regions. At the higher potential, the transition between the neutral DMBQ molecule and the radical anion species was considered to proceed (Scheme 1). Moreover, the lower potential region could reflect the transition between the radical anion state and the dianion state.

**Figure 3:**
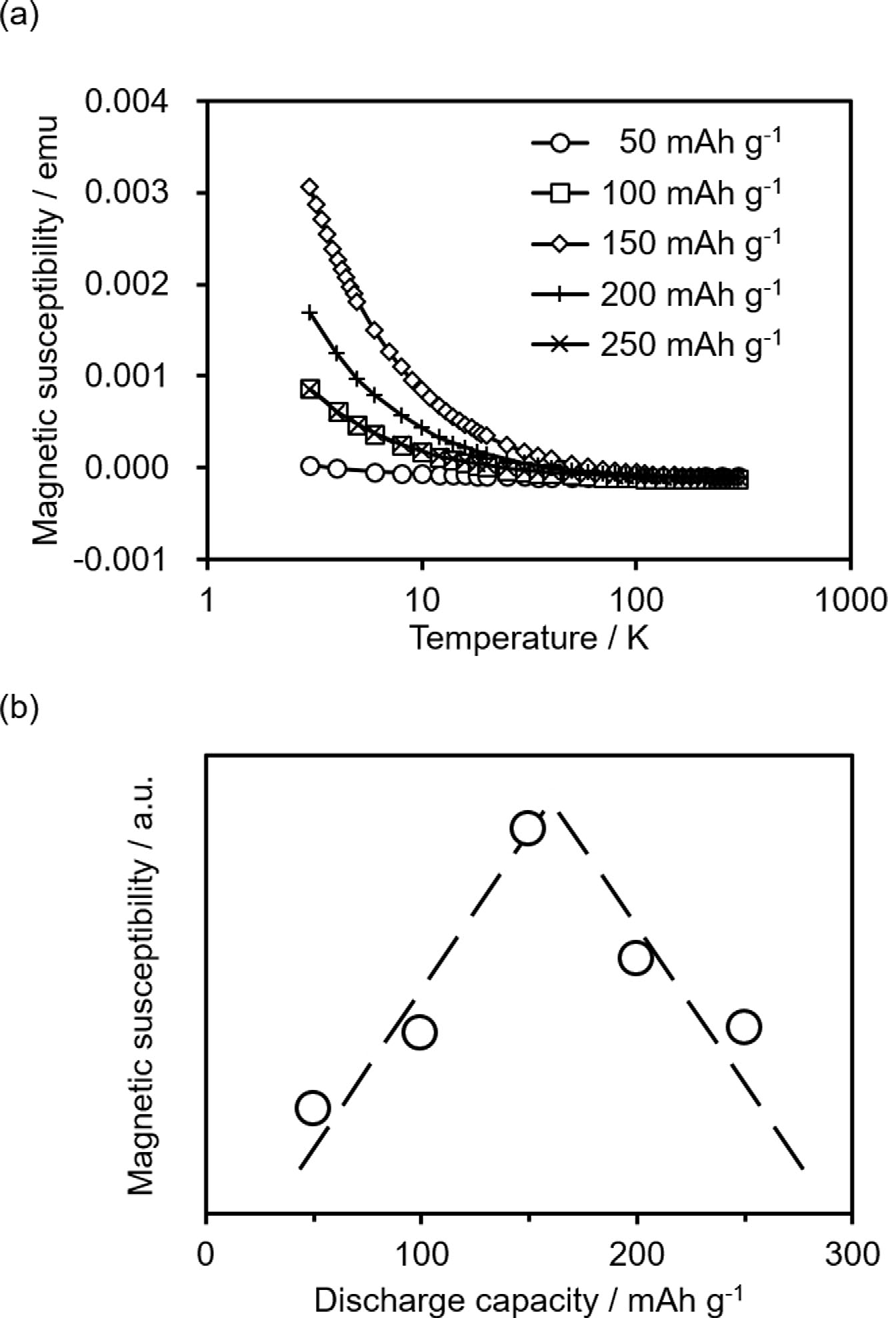
a) Temperature dependence of the magnetic susceptibility (magnetic susceptibility) at each discharge capacity. The temperature range was 3–300 K and the applied field was 0.5 T. b) Relationship between magnetic susceptibility and discharge capacity at 3.0 K.

To confirm the formation of radical species, a magnetic measurement was performed for the electrodes obtained from the cells, which were stopped at a given depth of discharge (DOD). Whereas, in the organic battery field, electron spin resonance (ESR) spectroscopy has often been applied for such a purpose, we decided to use another technique, the SQUID apparatus, in this study. SQUID, which specializes in measuring solid‐state samples, is an easy and powerful tool for obtaining a detailed temperature dependency and magnetic force dependency for magnetic susceptibility.^73^ Therefore, SQUID is commonly used in the molecular magnetism field to examine the magnetic interactions of solid samples. It is possible to amplify the magnetic responses from samples by increasing applied magnetic field strength. Accordingly, this technique is suitable for detecting a weak signal. Figure 3 a shows the temperature dependency of magnetic response (magnetic susceptibility) at each discharge capacity.

As expected, all samples showed a magnetic response that obeyed Curie's law [Eq. (1)]:

(1) χ=Const./T

where *χ* is magnetic susceptibility and *T* is absolute temperature in the applied temperature range. The observed behaviors were paramagnetic. This result indicates that the magnetic interaction between each molecule in the electrode was very weak, implying the high stability of radial species in the electrode. Furthermore, the intensity change of the magnetic response during the discharge process was examined at 3.0 K (Figure 3 b). The intensity of the magnetic response increased as the DOD increased to around 50 %, i.e., 150 mAh g^−1^. After the maximum intensity at approximately 150 mAh g^−1^, the response started to decrease. This observation indicates that, during the former discharge process, the radical concentration increased (DOD=0–50 %). It decreased in the subsequent discharge process (DOD=50–100 %), confirming the reaction mechanism model depicted in the Scheme 1.

### Operando Raman spectroscopy with DFT calculations

**Figure 4:**
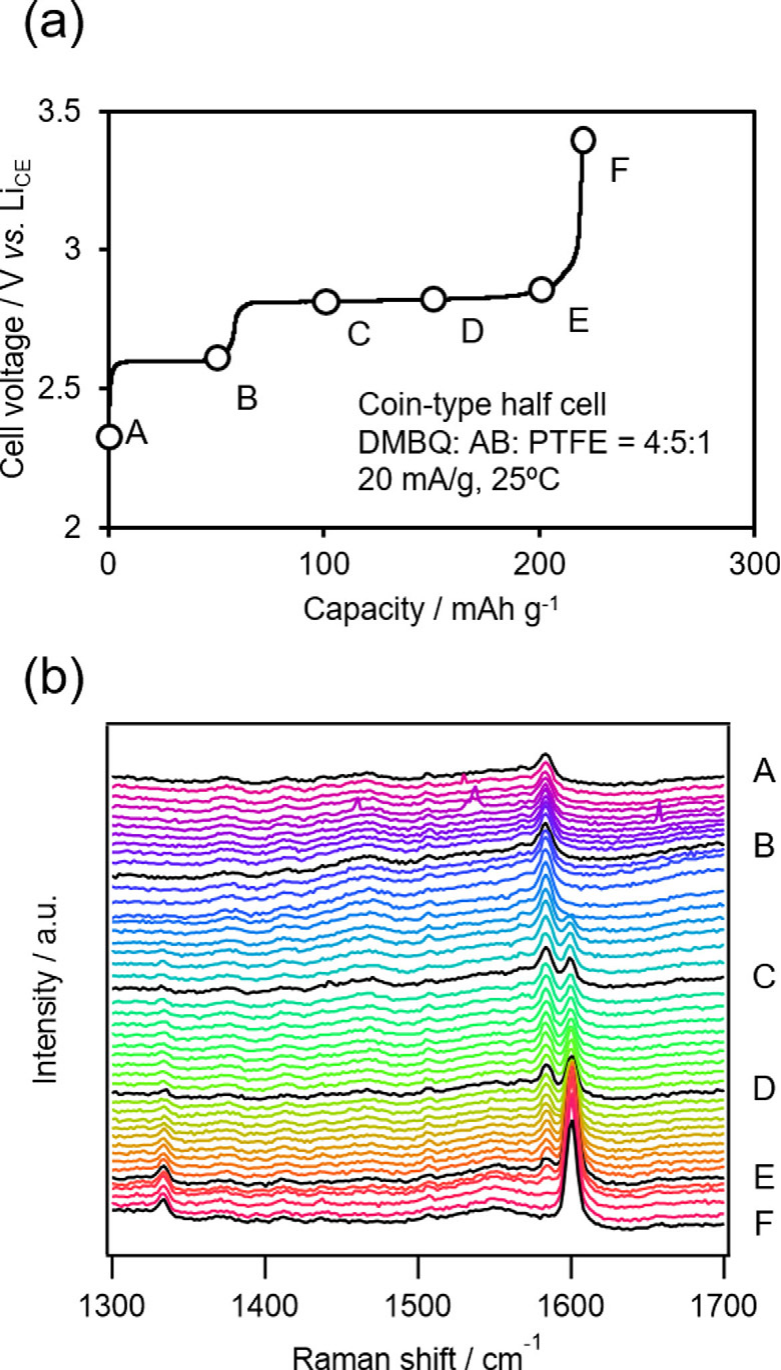
a) Second charge curve of the DMBQ half‐cell. The vertical axis shows the voltage between the DMBQ working electrode and the Li counter electrode (Li_CE_). b) Operando Raman spectra obtained at the circled points in (a).

We assumed that DMBQ changed its state, as shown in Scheme 1, during charge–discharge. The Raman spectra of each state should have been different from each other. Therefore, we performed Raman spectroscopy in operando while operating the cell. Figure 4 a shows the second charge curve of the DMBQ−Li half‐cell for the operando Raman spectroscopy. The spectra in black in Figure 4 b (labeled A–F) correspond to timings marked with circles in Figure 4 a.

During the lower plateau, there seems to have been no change in the Raman spectra. Note that the Raman spectrum obtained at state A may not have reflected the very state of A. The length of the lower plateau was lesser than that of the higher plateau, which is possibly because of the insufficient discharge during the last charging, which can be improved by applying a lower current density. During the upper plateau, there seems to have been changes at around 1334, 1584, and 1600 cm^−1^; the peaks at around 1334 and 1600 cm^−1^ increased, whereas that at around 1584 cm^−1^ decreased. These changes between the spectra indicate that a fraction of the main component in state B decreased to zero and a fraction of the main component in state F increased from zero during the upper plateau, which may indicate that there was a two‐phase coexistence at the upper plateau.

By using ex situ Raman spectroscopy, the differences in the Raman spectra during charge–discharge would have been difficult to analyze, largely because subtle differences in the conductive additives, binders, and impurities would have affected the spectra. However, operando Raman spectroscopy avoided such effects. Therefore, we were able to shed light on the DMBQ phase change analysis.

**Figure 5:**
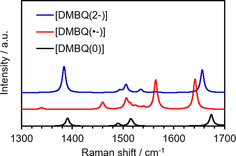
Simulated Raman spectra for the electrochemical states of the neutral state [DMBQ(0)], the radical monoanion state [DMBQ(⋅−)], and the dianion state [DMBQ(2−)] by using DFT at the B3LYP/6‐31+G(d) level.

Next, we considered that Raman spectroscopy alongside DFT calculations would help to examine the DMBQ phase change during charge–discharge. We performed DFT calculations to obtain the theoretical Raman shifts for certain states of DMBQ. The harmonic vibrational frequencies of the Raman spectra of [DMBQ(0)], [DMBQ(⋅−)], and [DMBQ(2−)] were calculated by using DFT at the B3LYP/6‐31+G(d) level in the Gaussian 16^74^ program and Gauss View 6^75^ molecular visualization program package. For the [DMBQ(⋅−)] and [DMBQ(2−)] states, the DMBQ skeleton was positioned next to one and two Li ions, respectively. These structures were then optimized. Figure 5 shows the obtained spectra. The simulation results were consistent with the measured results for the states from [DMBQ(⋅−)] to [DMBQ(0)], which correspond to the states from B to F. The simulation results were not consistent with the measured results for [DMBQ(2−)], which corresponds to state A. As mentioned above, the Raman spectroscopy must be conducted at a lower rate to afford more detailed discussion. The simulated peaks at 1393 cm^−1^ for [DMBQ(0)], 1645 cm^−1^ for [DMBQ(⋅−)], and 1659 cm^−1^ for [DMBQ(0)], all of which are related to skeleton plane vibration modes, are considered to correspond to the peaks at 1334 cm^−1^ for F, 1584 cm^−1^ for B, and 1600 cm^−1^ for F, obtained from the measured results.

### Crystallographic phase change examination by operando XRD

**Figure 6:**
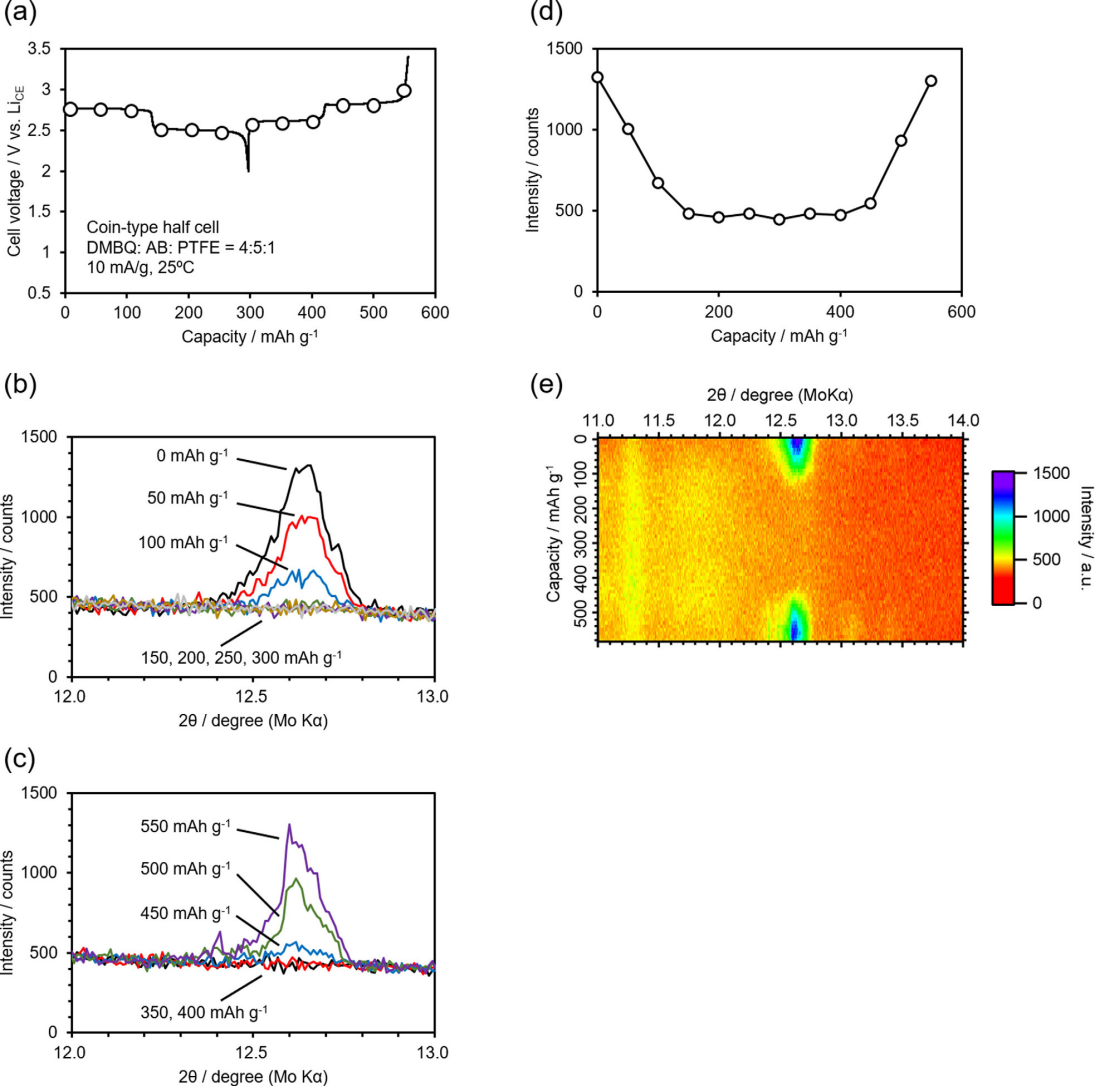
a) The initial discharge and the following charge curves of the DMBQ electrode for the operando two‐electrode coin cell. b, c) The XRD pattern of the DMBQ electrode obtained in operando condition during the initial discharge (b) and charge (c) at the moment indicated by circles in (a). d) Relationship between the peak intensity at around 12.5° in the XRD pattern and the discharge and charge capacities. e) XRD pattern of the DMBQ electrode obtained under operando conditions during initial discharge and charge.

As shown in Figure 1, the XRD pattern of the DMBQ electrode at the fully charged (delithiated) state had certain peaks derived from DMBQ in the crystalline state. However, the DMBQ electrode at the fully discharged (lithiated) state did not show peaks, indicating that it had lost long‐distance ordering.^61^, ^62^ In this case, the crystallographic information on DMBQ's transition state—the radical monoanion state [DMBQ(⋅−)]—was unclear. Thus, in this study, operando XRD measurements were carried out to reveal the transition state. Figure 6 a shows the initial discharge curve and the following charge curve of the DMBQ electrode using the operando two‐electrode coin cell. The curves have two plateaus and the capacity of each plateau was approximately 150 mAh g^−1^. The cell voltages at each plateau were roughly 2.8 and 2.6 V. These values closely resemble those reported previously,^62^ indicating that the operando cell is sufficient for examining DMBQ as a half‐cell and for XRD measurement. Figure 6 b, c, e shows XRD patterns of the DMBQ electrode obtained in operando condition during the initial charge–discharge. The XRD patterns shown in the Figure 6 b, c corresponds to the circled timing in Figure 6 a.

The discharged state of DMBQ is amorphous. This is evident because XRD patterns have no peaks other than the additives and current collector (see Figure 1). Figure 6b, c, e shows the gradual peak disappearance during the higher plateau in the initial discharge. No changes in the peak position or half width of the peak are observed, indicating the unchanged lattice constant of the existing crystal phase and unchanged crystallite size and, hence, two‐phase coexistence. If the reaction had been in the homogeneous phase, the peak would have gradually shifted and the half width might have changed during charge–discharge.

These changes indicate that long‐distance ordering gradually disappeared at a higher plateau. The phase then changed to an amorphous one. For the XRD profile change during the lower plateau in the initial discharge, no change in the pattern and thus no long‐distance ordering was observed. With reference to the lower plateau region, no XRD profile changes were observed, even during the subsequent charge. However, the peak that had disappeared returned when the charging process proceeded to the higher plateau region. The peak intensity gradually increased without any changes in the half width or peak position, indicating that this transition system was a two‐phase coexistence one.

The relationship between the peak intensity at around 12.5° in the XRD pattern and the discharge and charge capacity is shown in Figure 6 b. Regarding the peak intensity during the higher plateau, there was an increase and decrease according to the state of charge.

Poizot et al.^12^ observed similar two‐phase coexistence behavior by using the galvanostatic intermittent titration technique for another DMBQ analog, 2,5‐diamino‐1,4‐benzoquinone (DABQ), which gave rise to very flat plateaus. Moreover, they reported that the operando XRD pattern of DABQ directly indicated two‐phase coexistence.^12^ In their study, DABQ had long‐distance ordering in the fully charged state, the half‐charged state, and the fully discharged state. This is a different result to that obtained for DMBQ, and the origin of this difference requires clarification in the future.

**Figure 7:**
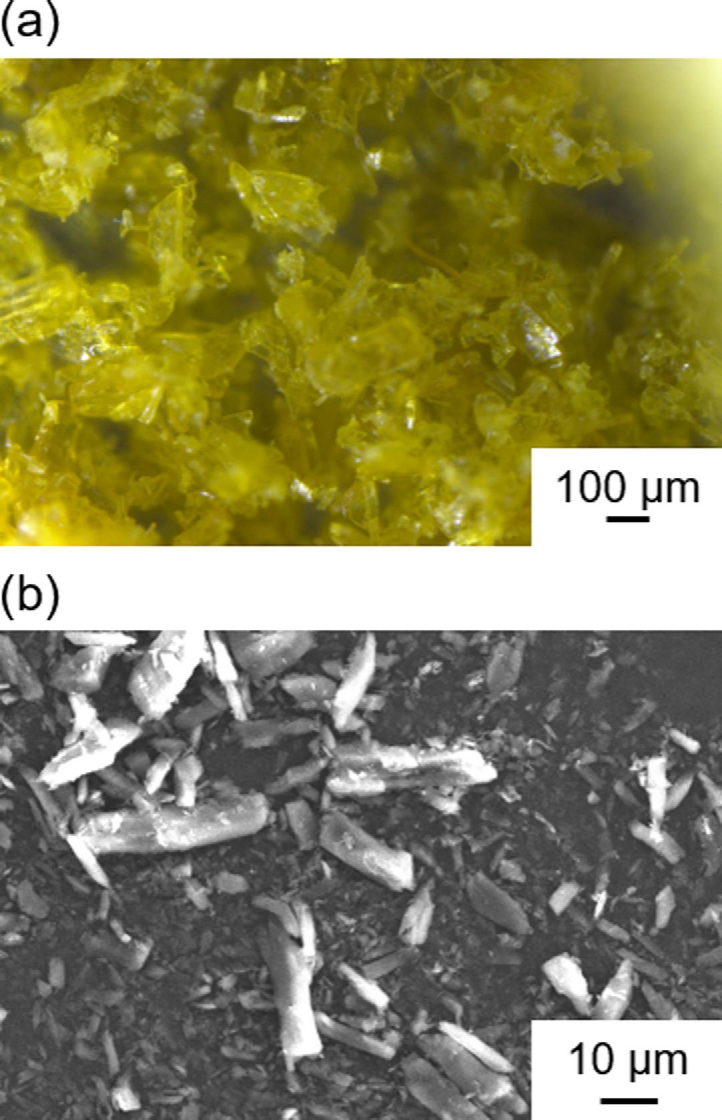
a) Optical and b) SEM images of the DMBQ powder.

In this study, the peaks at 2*θ*≈12.5° were investigated. Figure 7 shows the optical and scanning electron microscopy (SEM) images of the DMBQ powder before electrode fabrication. From these images, the powder appears to be in a crystal phase, which is confirmed by the powder XRD pattern of DMBQ (Figure 1).

**Figure 8:**
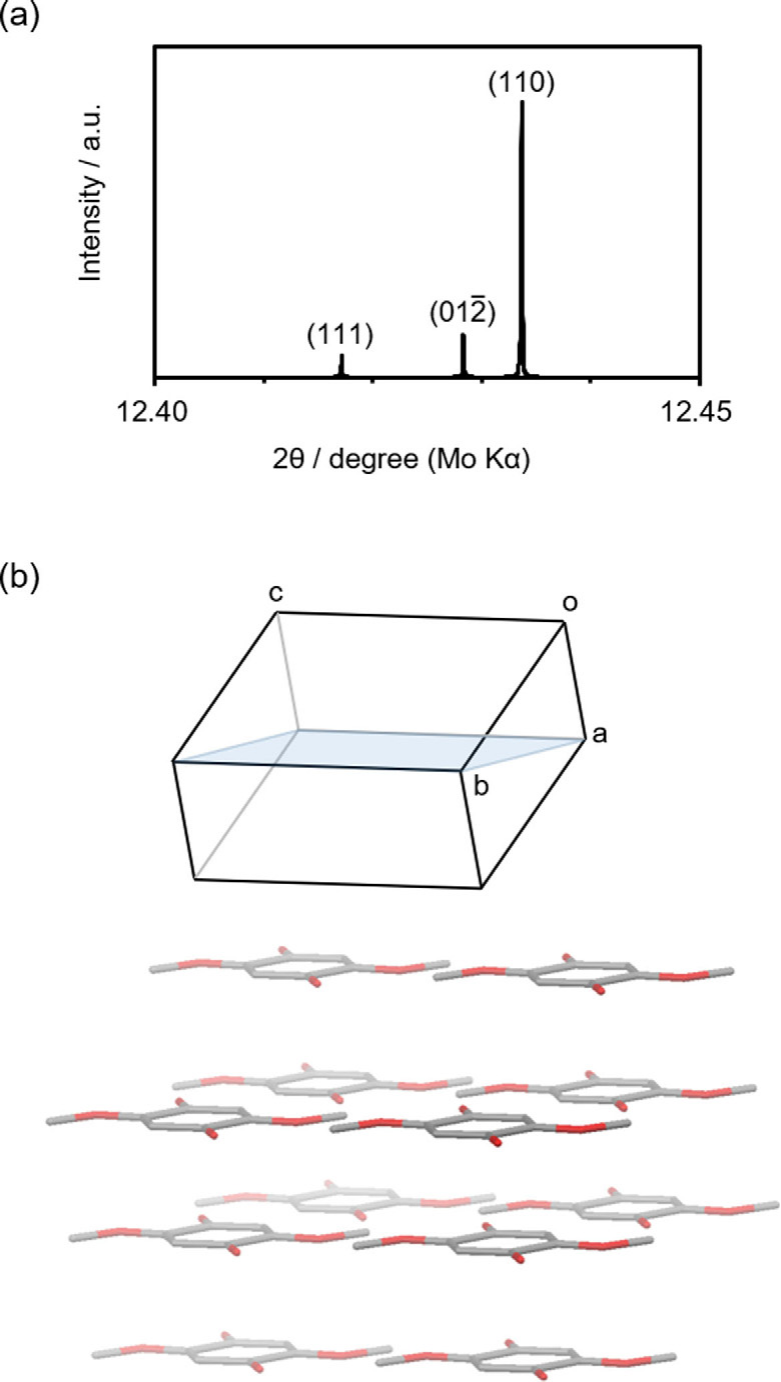
a) Simulated XRD pattern for DMBQ with the reported crystallographic data.^76^ The half width is 0.001°, the step is 0.001°, and the light source is MoKα. b) Crystal image of the DMBQ molecule arrangement with the π‐stack direction.

Figure 8 shows the XRD pattern for DMBQ simulated by using the reported crystallographic data^76^ and a molecular arrangement in the crystal. From the crystallographic data, the peak at approximately 12.5° is derived from the scattering in the (110), (01−2), and/or (111) directions. When the crystal lacks strong anisotropy, it indicates that the peak at around 12.5° comes mostly from the scattering in the (110) direction. One of the (110) planes is shown in light blue in Figure 8 b.

### Na system

#### Elucidation of the carrier ion

**Figure 9:**
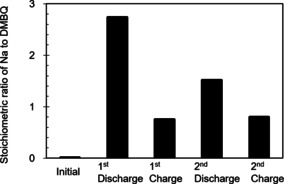
The change in stoichiometric ratio of Na to DMBQ (*R*_Na/DMBQ_) obtained from the EDX measurements in the first two cycles.

We next examined DMBQ in a Na system. Many organic cathodes have been reported to function both in Li systems and in Na systems, as well as other multivalent‐cation‐based systems.^6^ To identify the charge carrier in the DMBQ battery for the Na system, the Na content concentration change in the electrode was examined ex situ by using the energy dispersive X‐ray spectroscopy (EDX; Figure 9). Whereas the pristine electrode had no Na, the stoichiometric ratio of Na to DMBQ (*R*_Na/DMBQ_) increased to 2.7 after the first discharge. During the subsequent first charge, the ratio decreased to 0.7. Although the obtained values during the initial charge–discharge process seemed to have been slightly overestimated compared with the redox model, in which the ratio is supposed to change between 0 and 2, the value difference agrees with the model very well. This tendency was repeated for the second cycle, although the rate of change became lesser, thus reflecting the capacity decay during cycling. The observed stoichiometric ratio change indicates that the DMBQ molecule receives and releases Na ions during the cathodic and anodic reactions as described above, confirming that the charge carrier in this system is certainly the Na ion.

### Crystallographic phase change examination for the Na system

**Figure 10:**
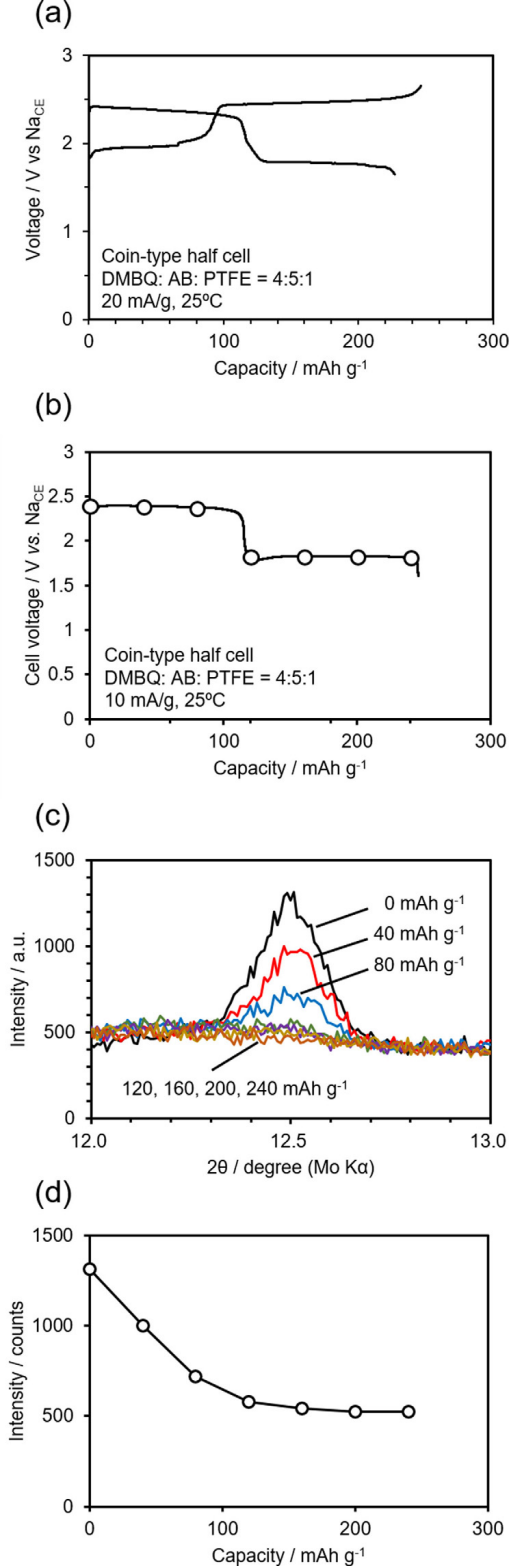
a) The initial discharge and the following charge curves of the DMBQ electrode in a Na system with a traditional two‐electrode coin cell. The vertical axis shows the voltage between the DMBQ working electrode and the Na counter electrode (Na_CE_). b) The XRD pattern of the DMBQ electrode obtained under operando conditions during initial discharge. c) Initial discharge curves of the DMBQ electrode in a Na system with a two‐electrode coin cell for operando measurements. The circled point indicates the time when the XRD patterns were obtained under operando conditions. d) Relationship between the peak intensity at approximately 12.5° in the XRD pattern and the discharge capacity.

The phase change in crystallography was not as clear for the Na system as for the Li system. Therefore, we first assembled the conventional coin cell with DMBQ in the Na system. Figure 10 a shows the first discharge and the following charge curve. The obtained capacity was approximately 240 mAh g^−1^, which is equivalent to roughly 80 % of the theoretical capacity, assuming the two‐electron redox reaction of DMBQ. Thus, we were able to confirm that DMBQ definitely also works for the Na system. Figure 10 c shows the XRD pattern of the electrode obtained in operando conditions during the initial discharge obtained at the points marked with circles in Figure 10 b. Figure 10 d shows the variation in peak intensity in the XRD pattern shown in Figure 10 c. The changes in the XRD pattern were very similar to those for the Li system. In future, we aim to develop this type of experiment for a Mg system,^43^ in which the monovalent DMBQ would be in a different state compared to the multivalent counter cation.

## Conclusions

In this study, we investigated, by using several tools, the structural change of DMBQ when it is used as an active material. ^7^Li NMR and EDX spectroscopies demonstrated that the DMBQ molecules receive and release Li ions and Na ions, in the Li system and Na system, respectively, during the cathodic and anodic reactions. Moreover, these tools revealed that the charge carriers were the Li ion and the Na ion, respectively. Magnetic measurements by using SQUID and operando Raman spectroscopy alongside DFT calculations produced data supporting the premise that DMBQ molecules in the electrode adopt three states during charge–discharge, whereas the intermediate state is a radical species.

Operando XRD, which is a very simple method in which a hole is punched out from the cell exterior, was applied to the crystallographic analysis of DMBQ. Consequently, the DMBQ sample was considered to be in a two‐phase coexistence state with the neutral [DMBQ(0)] state and the radical monoanion [DMBQ(⋅−)] state at the higher plateau. Moreover, the radical monoanion [DMBQ(⋅−)] and dianion [DMBQ(2−)] phases showed no long‐distance ordering for either Li or Na systems. Two‐phase coexistence during charge–discharge was also reported for other quinone derivatives.^4^, ^9^, ^12^, ^15^, ^18^ Furthermore, the measurements revealed that these structures without long‐distance ordering reversibly return to the original neutral [DMBQ(0)] phase with long‐distance ordering. In future, the crystallographic information on the [DMBQ(⋅−)] and [DMBQ(2−)] phased on short‐range ordering should be examined by using suitable techniques such as X‐ray absorption fine structure spectroscopy. Interestingly, as confirmed by this and previous studies, DMBQ works in a divalent Mg system.^43^, ^45^, ^47^ DMBQ crystallographic examination during charge–discharge in a divalent Mg system is of considerable interest and requires further examination. The techniques explained herein are helpful for revealing the charge–discharge mechanism and factors determining the deterioration of organic batteries. This will be useful as a guide when designing high‐performance organic batteries in future.

## Experimental Section

### Electrode preparation

A positive electrode composite sheet was first prepared by mixing DMBQ powder (>98.0 %, Tokyo Chemical Industry, Japan), acetylene black as the conductive additive, and poly(tetrafluoroethylene) (PTFE) as the binder, in the weight ratio 4:5:1. The sheet was then pressed onto a mesh‐type stainless steel current collector. The electrode was dried under vacuum at 60 °C for 1 h.

### Cell fabrication

Cells were fabricated in an argon‐filled glove box. A two‐electrode coin‐type cell was fabricated in a dry chamber, in accordance with a reported method^65^ where the dew point was <−60 °C. The positive electrode composite sheet pressed onto a mesh‐type stainless steel current collector, Li foil, 1 m LiClO_4_/γ‐butyrolactone (GBL), and a glass fiber filter were used as the working electrode, the counter electrode, the electrolyte, and the separator, respectively. The aforementioned process was for the Li system, whereas Na foil and 1 m NaClO_4_/GBL were used as the counter electrode and the electrolyte, respectively, for the Na system.

### Electrochemical treatment

DMBQ, and thus the cells, were in a charged state initially. Thus, the current first flowed in the discharge direction. The cell was then discharged and charged at room temperature. The current density and cut‐off voltage range was set to be 10–20 mA g_(DMBQ)_^−1^ and 2.0–3.4 V for the Li system, and 1.65–2.65 V for the Na system. In this study, the obtained capacities are expressed per mass of DMBQ. For the ex situ experiment, the electrochemical treatment was stopped, for example, just after the first discharge and the following charge.

### Operando XRD measurements and cell fabrication

**Figure 11:**
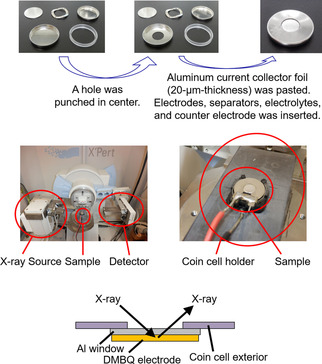
Photographs and schematic representation of the cross section of the coin cell and the XRD machine used for operando XRD measurements.^65^

A two‐electrode coin‐type cell was fabricated in the same manner as described above. For X‐rays to penetrate through the exterior, a hole was punched in the center of the working electrode side of the exterior. Then, Al foil, with a 20 μm thickness, was pasted at the hole, and the gap was sealed with vacuum grease (Figure 11). Each cell was then set in the XRD machine to analyze the change in the bulk structure of DMBQ on discharge and charge using an X′Pert PRO MPD (PANalytical, Netherlands) diffractometer with MoKα radiation (wavelength, *λ*=0.70932 Å) at an acceleration voltage of 60 kV and a current density of 50 mA (Figure 11). A MoKα line was used to reduce the attenuation of the Al foil rather than the copper radiation source. The XRD scans ranged from 2*θ*=11°– 14°, with a step size of 0.008°. Each scan took 5 min at room temperature. We then collected the operando XRD data of the DMBQ−Li system and the DMBQ−Na system by using the deintercalation process (electrochemical oxidation process) and intercalation process (electrochemical reduction process).

### Operando Raman spectroscopy and cell fabrication

**Figure 12:**
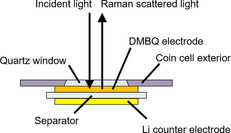
Schematic representation of the cross‐section of the coin cell used for operando Raman spectroscopy.

A two‐electrode coin‐type cell was fabricated in the same manner as described above. Figure 12 shows the structure of the cell used in this measurement. For light to penetrate through the exterior, a quartz glass window was positioned in the center of the working electrode side of the exterior. Note that the mixed electrode material was pressed into an Al mesh instead of onto metal foil such that the Raman scattered light could be detected from the side without a counter electrode. The Raman spectrum was then obtained in operando for each cell by using a Raman microscope (RAMANtouch VIS‐NIR‐LT, Nanophoton, Japan). The wavelength was 532 nm. The spectrum was obtained by averaging the signal from the scanned area (area flash mode in the machine) to prevent light energy from being concentrated in the focused area. The area was approximately 1500×1500 μm. The exposure time was 10 s (10 times of 1 second) for one spectrum and for one scanned area.

### Ex situ XRD measurements

The same set‐up was used as that used for the operando XRD measurements, but a Cu radiation source was used rather than a Mo X‐ray source. After disassembling the cell without holes, the electrodes were taken from the cells and washed with GBL to remove the electrolyte salt. The resulting data for these measurements is shown in Figure 1.

### Energy dispersive X‐ray spectroscopy

EDX measurements were conducted *ex situ* for the quantitative analysis of elements in the electrode. In the experiment, the cells were disassembled in an argon‐filled glove box. The DMBQ electrodes taken from the cells were wiped to remove the electrolyte solution and were then dried in a vacuum. The samples were then transferred to the chamber of a SEM (JSM‐IT100, JEOL, Japan). The atomic ratios of sodium, fluorine, and chlorine were then collected, and then the values were corrected to remove the influence of the residue of the electrolyte salt (NaClO_4_) by using the chlorine ratio. The calculated stoichiometric ratios of Na to F (*R*_Na/F_) were 1.6, 0.4, 0.9, and 0.5 for the first discharge, first charge, second discharge, and second charge samples, respectively. Then, these values were converted to the stoichiometric value of Na to DMBQ (*R*_Na/DMBQ_), considering the molar ratio of PTFE and DMBQ in the electrode.

### ^7^Li NMR spectroscopy

The Li ion concentration in the electrode during the charge–discharge process was measured ex situ by ^7^Li NMR spectroscopy (500 MHz, JNM‐ECA series, JEOL, Japan). For this measurement, the cells were assembled with a tetrahydrofuran‐based electrolyte solution. The electrodes were taken out of the cells after a given charge–discharge process and were rinsed with a pure solvent to remove the electrolyte salt. The water‐soluble contents were thoroughly extracted from the electrodes by immersing each electrode in a given amount of deuterium oxide. For this measurement, a coaxial double tube was used. The internal tube was filled with an organic solution of lithium bis(trifluoromethanesulfonyl)imide to use the signal intensity as an internal standard. The chemical shift values for the ^7^Li nuclei in water and an organic solvent were different, reflecting the environmental difference. Therefore, it should be possible to detect the ^7^Li nuclei intensity change in the target samples. The relative Li intensities of these solutions were first obtained using the ^7^Li nucleus signal intensity in the solutions by comparing these signals with those of the internal Li standard solution. These values (ca. 1.4, 0.3, 1.3, and 0.2 for the first discharge, first charge, second discharge, and second charge samples, respectively) were altered to the quantitative concentration values of each solution by using calibration curves made in advance. Finally, the obtained values were converted to the stoichiometric ratio of Li to DMBQ (*R*_Li/DMBQ_) in the electrodes.

### Magnetic measurement using SQUID

The cells that were stopped at a given state of discharge (50, 100, 150, 200, 250 mAh g^−1^) were disassembled. After removing the residual electrolyte solutions, the sample electrodes were then dried under vacuum and packed in plastic capsules. The magnetic properties of sample electrodes were measured by a SQUID magnetometer (Quantum Design) at a magnetic field strength of 0.5 T in the temperature range of 3–300 K.^73^

### DFT calculations for Raman shift prediction

The harmonic vibrational frequencies of the Raman spectra of [DMBQ(0)], [DMBQ(⋅−)] and [DMBQ(2−)] were calculated by DFT at the B3LYP/6‐31+ G(d) level using the Gaussian 16^74^ program and the Gauss View 6^75^ molecular visualization program package. For the [DMBQ(⋅−)] and [DMBQ(2−)] states, the DMBQ skeleton was positioned next to one and two Li ions, respectively, and the structures were optimized.

## Conflict of interest

*The authors declare no conflict of interest*.
